# High-Grade Progression, Sarcomatous Transformation, and/or Metastasis of Pituitary Neuroendocrine Neoplasms (PitNENs): The UCSF Experience

**DOI:** 10.1007/s12022-024-09829-w

**Published:** 2024-10-10

**Authors:** Merryl Terry, Minh P. Nguyen, Vivian Tang, Ekin Guney, Krishna L. Bharani, Sonika Dahiya, Ondrej Choutka, Ewa Borys, Gerald Reis, Lewis Blevins, Manish K. Aghi, Sandeep Kunwar, John DeGroot, David R. Raleigh, Melike Pekmezci, Andrew W. Bollen, Soonmee Cha, Nancy M. Joseph, Arie Perry

**Affiliations:** 1 Department of Pathology, Division of Neuropathology, University of California San Francisco, San Francisco, CA USA; 2 Department of Pathology, Stanford University, Palo Alto, CA USA; 3 Department of Pathology, Washington University, St. Louis, MO USA; 4 Department of Neurosurgery, Saint Alphonsus Neuroscience Institute, Boise, ID USA; 5 Department of Pathology, Loyola University, Chicago, IL USA; 6 Department of Pathology, Memorial Healthcare System, Hollywood, FL USA; 7 Department of Endocrinology, University of California San Francisco, San Francisco, CA USA; 8 Department of Neurological Surgery, University of California San Francisco, San Francisco, CA USA; 9 Department of Radiation Oncology, University of California San Francisco, San Francisco, CA USA; 10 Department of Radiology, University of California San Francisco, San Francisco, CA USA

**Keywords:** Pituitary carcinoma, Pituitary neuroendocrine carcinoma, Pituitary neuroendocrine tumor, Pituitary sarcoma, Grading, Metastasis, Neuroendocrine neoplasms

## Abstract

Pituitary neuroendocrine tumors (PitNET) that metastasize comprise ~ 0.2% of adenohypophyseal tumors are aggressive and are challenging to treat. However, many non-metastatic tumors are also aggressive. Herein, we review 21 specimens from 13 patients at UCSF with metastatic PitNETs (CSF or systemic, *N* = 7 patients), high-grade pituitary neuroendocrine neoplasms (HG-PitNEN, *N* = 4 patients), and/or PitNETs with sarcomatous transformation (PitNET-ST, *N* = 5 patients). We subtyped cases using the World Health Organization (WHO) and International Agency for Research on Cancer (IARC) criteria for neuroendocrine neoplasms (NENs). Lineage subtypes included acidophil stem cell, null cell, thyrotroph, corticotroph, lactotroph, and gonadotroph tumors. The median Ki-67 labeling index was 25% (range 5–70%). Lack of p16 was seen in 3 cases, with overexpression in 2. Strong diffuse p53 immunopositivity was present in 3 specimens from 2 patients. Loss of Rb expression was seen in 2 cases, with ATRX loss in one. Molecular analysis in 4 tumors variably revealed *TERT* alterations, homozygous *CDKN2A* deletion, aneuploidy, and mutations in *PTEN*, *TP53*, *PDGFRB*, and/or *PIK3CA*. Eight patients (62%) died of disease, 4 were alive at the last follow-up, and 1 was lost to the follow-up. All primary tumors had worrisome features, including aggressive lineage subtype, high mitotic count, and/or high Ki-67 indices. Additional evidence of high-grade progression included immunohistochemical loss of neuroendocrine, transcription factor, and/or hormone markers. We conclude that metastatic PitNET is not the only high-grade form of pituitary NEN. If further confirmed, these histopathologic and/or molecular features could provide advanced warning of biological aggressiveness and be applied towards a future grading scheme.

## Introduction

The term “pituitary adenoma” was recently modified to the pituitary neuroendocrine tumor (PitNET) to align with the nomenclature now used for systemic neuroendocrine neoplasms (NENs). This was also done to communicate their malignant potential more clearly. PitNETs are well-differentiated neuroendocrine neoplasms of the anterior pituitary and present with a wide range of clinical, radiologic, and histopathological features. PitNETs are common (identified incidentally in up to 20% of the population [1]) and comprise ~ 16% of all brain tumors in the USA [2]. Often considered “benign” as a group, ~ 7–35% are nonetheless clinically aggressive, being associated with excess morbidity and mortality [3–6]. As such, aggressive PitNETs should be considered tumors with at least some malignant potential despite the rarity of metastasis. Both aggressive PitNETs and metastatic PitNETs are now known to be associated with premature death [7].

Despite the wide biological variability encountered clinically, predicting PitNET behavior using routine histopathology alone remains elusive [8]. There is currently no formal grading system for PitNETs and the current WHO classification system (Endocrine 2022) places PitNETs into one of two major categories: PitNET (with various subtypes) and metastatic PitNET. There is currently no well-defined intermediate group, although a few of the lineage-based subtypes are thought to be innately more aggressive. The previously utilized term of “atypical adenoma” has been abandoned in both the Endocrine and CNS WHO classification schemes due to a lack of definitional reproducibility and prognostic significance. The term “metastatic PitNET” has replaced the term “pituitary carcinoma,” requiring evidence of craniospinal dissemination and/or systemic metastases. This new terminology better reflects the fact that most metastatic PitNETs remain well-differentiated even in metastatic deposits. What’s more, given that “metastatic adenoma” is a biological oxymoron, this terminology also avoids the awkward nomenclature transition from a previously diagnosed “adenoma” into a full-blown malignancy (i.e., pituitary carcinoma). “Pituitary neuroendocrine carcinoma” (PitNEC) is a relatively newly proposed term aligning with the 2018 IARC guidelines for grading systemic NENs elsewhere [9] and is reserved for exceptionally rare poorly differentiated PitNENs with unusually high Ki-67 labeling indices and/or mitotic counts, as well as histopathology resembling small or large cell carcinoma. If the IARC grading scheme were to be applied to PitNENs, a small subset of tumors might reach grades 2, 3, or rarely PitNEC, potentially alerting the clinical team upfront of the increased possibility of biological aggressiveness. Within such a scheme, high-grade tumors (i.e., PitNET grade 3 or PitNEC) are considered exceptional [10]. In fact, they are so rare that controversy remains around whether high-grade progression of PitNETs even exists.

Additional attempts have been made at predicting which individual PitNETs will behave aggressively. As previously mentioned, there are several reproducible and well-documented prognostic associations with lineage-associated subtyping of PitNETs [11], particularly those with immature or less well-differentiated cytomorphology. However, some of these aggressive subtypes are sufficiently rare that associations with clinical behavior are not fully established yet. Nevertheless, lineage-associated PitNET subtyping is currently recommended by both the Endocrine and CNS WHO schemes. Other prognostic strategies include integrating clinical and radiologic data with histopathologic and immunohistochemical findings. Radiologic definition of tumor aggressiveness includes the Knosp classification scheme [12], which stratifies tumors by their degree of lateral extension into the cavernous sinuses, and the Hardy classification scheme, which assesses the degree of extra-sellar and vertical extension [13]. Trouillas et al. proposed a clinicopathologic grading scheme which has been validated in multiple studies, showing statistically significant associations with tumor recurrence [6, 14–16]. It uses a combination of radiologic findings, p53 positivity, and Ki-67 index to stratify tumors into five clinicopathologic “grades” which range from non-invasive tumors with low Ki-67 labeling (grade 1a) to metastatic tumors (grade 3). Of particular interest is the “grade 2b” category (radiologically invasive PitNETs with high proliferation), which overlaps partially with the European Society of Endocrinology (ESE) definition of a locally aggressive PitNET. Grade 2b tumors had a poor prognosis with an increased probability of tumor persistence or subsequent progression. At present, however, this prognostic scheme has not been widely adopted by pathologists. This is possibly due to the difficulties in applying sometimes subjective radiologic criteria for tumor invasion, proliferative cutoffs that are prone to interobserver variability and are probably too low, the general trend away from p53 immunostaining due to a lack of any independent prognostic value in PitNETs, and the frequency with which radiologic information is unavailable to the pathologist at the time of diagnosis.

To better understand the range of clinically aggressive tumors, we reviewed our experience at UCSF with metastatic PitNETs, high-grade PitNENs, and PitNETs with sarcomatous transformation, including 21 specimens from 13 patients. We analyzed their clinical, radiologic, histopathologic, immunohistochemical, and molecular features.

## Materials and Methods

This study was approved using the Institutional Review Board, Human Research Protection Program Committee on Human Research, protocol 10–03204. We performed a text query of the electronic pathology database for the term “pituitary carcinoma” on in-house and consulted neuropathology cases at UCSF. Archival specimens were retrieved from the anatomic pathology files and slides were reviewed for diagnostic accuracy. Older cases with no available tissue or limited records were excluded. Glass slides and tissue blocks were retrieved from the remaining cases. In addition, we performed a search of stored histologic and radiographic images from author AP. Patients 8 and 10 were previously reported [17, 18]. Additionally, patient 6 in this series is presented as case 4 in the paired submission by Joseph et al. on the high-grade progression of low-grade NETs.

Demographic and clinical data were obtained from a review of the electronic medical record. When available, diagnostic imaging was also reviewed. Mortality data and cause of death were extracted from the electronic medical record and supplemented from the outcomes data in the UCSF Cancer Registry. Overall survival was defined as the length of time spanning initial diagnosis until death. Survival time from the development of metastases, high-grade progression, and/or sarcomatous transformation until death was also calculated (“MATS” in Table 1). In a subset of cases, missing individual immunohistochemical stains were also performed on whole sections (e.g., pituitary hormones or transcription factors).

**Table 1.**
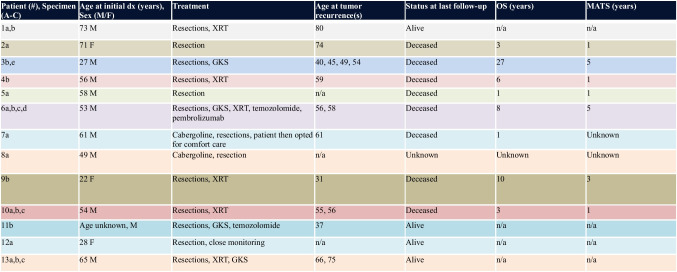
Patient demographics, treatment, and survival

*GKS* gamma-knife surgery, *OS* overall survival, *MATS* survival time from development of metastases or anaplastic (high-grade or sarcomatous) transformation until death, *n/a* not applicable, *XRT* external radiation therapy

A tissue microarray (TMA) was constructed for additional immunohistochemistry at UCSF. H&E-stained sections of select tumor cases were examined and representative areas of solid tumor were identified for sampling. Three 2-mm punch biopsy tissue cores per donor block were transferred to a recipient block using a Beecher Manual Tissue Microarray Machine (Sun Prairie, WI). Four micron-thick sections were cut from the TMA blocks and used for immunohistochemical stains.

Immunohistochemistry was performed on formalin-fixed, paraffin-embedded whole tissue sections or TMA sections using the following antibodies: p16 (Roche, clone E6H4, undiluted, 30-min antigen retrieval), TPIT (Sigma, clone CL6251, 1:500 dilution, 32-min antigen retrieval), PIT1 (Santa Cruz Biotech, clone D-7, 1:100 dilution, 30-min antigen retrieval), SF1 (R&D Systems, clone N1665, 1:100 dilution, 30-min antigen retrieval), ACTH (Agilent, clone 02A3, dilution 1:4000, 15-min antigen retrieval), human growth hormone (GH, Cell Marque, polyclonal, undiluted, 15-min antigen retrieval), prolactin (PRL, Cell Marque, polyclonal, 1:4 dilution, 15-min antigen retrieval), thyroid stimulating hormone (TSH, Leica, clone QB2/6, 1:400 dilution, 15-min antigen retrieval), CAM 5.2 (BD Biosciences, clone CAM5.2, 1:50 dilution, 30-min antigen retrieval), GATA3 (Roche, L50-823, undiluted, 60-min antigen retrieval), estrogen receptor (ER, Roche, clone SP1, undiluted, 28-min antigen retrieval), Rb (BD Biosciences, G3-245, 1:100 dilution, 30-min antigen retrieval), p53 (Leica, clone DO-7, undiluted, 30-min antigen retrieval), ATRX (Sigma, polyclonal, 1:100 dilution, 60-min antigen retrieval), anti-mitochondrial antigen (AMA, Biogenix Laboratories, clone 113–1, 1:500 dilution, 15-min antigen retrieval), Ki-67 (Agilent, clone MIB1, 1:50 dilution, 30-min antigen retrieval), synaptophysin (SPH, Cell Marque, polyclonal, 1:100 dilution, 30-min antigen retrieval), chromogranin (CHR, Cell Marque, clone LK2H10, 1:4 dilution, 15-min antigen retrieval), and insulinoma-associated antigen-1 (INSM1, Cell Marque, clone MRQ-70, undiluted, 52-min antigen retrieval). Immunostaining for TPIT, GATA3, ER, ATRX, and INSM1 was performed in a Ventana BenchMark Ultra automated stainer. All other stains were performed in a Leica BOND-III automated stainer. Diaminobenzidine was used as the detection chromogen, followed by a hematoxylin counterstain.

Immunohistochemistry for p16 was described as absent (no staining in tumor cells, but retained in non-neoplastic cells), retained (normal or wildtype, with patchy staining in tumor cells), or overexpressed (diffuse staining in tumor cells). Rb immunohistochemistry was interpreted as lost (complete lack of staining in tumor cells alongside a positive internal control such as vasculature) or retained (at least some staining in tumor nuclei). Overexpression of p53 was defined as strong nuclear staining in > 50% of tumor nuclei, which was considered a likely mutant pattern of protein expression; no cases with a null phenotype (i.e., all tumor nuclei negative) were encountered. Pituitary transcription factors (SF1, TPIT, PIT1) were interpreted as positive if strong nuclear staining was present in any fraction of tumor nuclei. Ki-67 labeling index was defined as the percentage of neoplastic cells which stained strongly for Ki-67 (minimum of 1000 cells counted) within tumor hotspots.

PitNETs were classified according to the CNS WHO 2021 and Endocrine WHO 2022 lineage and the 2018 IARC grading schemes for neuroendocrine neoplasms [19, 20]. Study cases included those with CSF or systemic metastases (metastatic PitNET), sarcomatous transformation (PitNET-ST), and/or high-grade PitNEN (corresponding to IARC PitNET grade 3 or PitNEC). PitNET subtyping was performed using Endocrine WHO 2022 guidelines based on immunohistochemical cell lineage and cytomorphology. PitNETs with sarcomatous transformation (PitNET-ST) were those with a spindled pattern, cytologic anaplasia, loss of cytokeratin, hormone and/or hormone/transcription factor staining, and immunopositivity for muscle markers and/or increased reticulin/type IV collagen deposition. The IARC scheme divides PitNENs into well-differentiated or poorly differentiated categories as follows: well-differentiated NETs are subdivided into grades 1, 2, and 3 (G1, G2, and G3) based on Ki67 indices of ≤ 3, 3–20, and > 20% and/or < 2, 2–20, and > 20 mitoses per 10 high-power fields (roughly 2 mm^2^), respectively. Neuroendocrine carcinomas (NECs) are defined as cytologically poorly differentiated neoplasms with > 20 mitoses/10 HPF and/or a Ki67 labeling index > 20%. The division between IARC PitNET grade 3 and PitNEC was challenging in several of our cases because the determination of features resembling large cell neuroendocrine carcinoma was subject to considerable interobserver variability. Therefore, we instead lumped these tumors into a single category of high-grade PitNEN (HG-PitNEN).

Targeted next-generation sequencing was performed using the UCSF500 cancer panel as previously described [21].

## Results

### Clinical Features

Clinical features are summarized in Table 1. We identified 21 specimens from 13 patients (10 male, 3 female) with a median age of 55 years at initial diagnosis (range 22–73). Four patients had a history of PitNET previously diagnosed at another hospital, who then presented to us at the time of recurrence. Five patients (patients 2, 5, 7, 8, and 12) had no known history of PitNET and underwent a single resection, though case 2 had been followed radiologically and case 12 had symptoms for several years prior to initial surgery. Patient 5 presented with a metastatic null cell PitNET (grade 2). Patient 7 presented with an acidophil stem cell tumor (HG-PitNEN), who opted for comfort care after significant growth despite cabergoline therapy. Patient 8 presented with a locally aggressive lactotroph PitNET with sarcomatous transformation (PitNET-ST). Patient 12 presented with Cushing syndrome and a corticotroph PitNET (borderline grades 2–3). The median time from initial diagnosis to recurrence was 3 years (range 1–13).

Four patients were alive at follow-up (31%), 8 patients were deceased (62%), and one patient was lost to the follow-up. In patients who died, the median overall survival time was 4.5 years (range 1–27) from initial diagnosis. The median time from high-grade/sarcomatous transformation and/or development of metastasis to death was 1 year (range 1–5 years). In patients who are still living, the interval from diagnosis to last known follow-up ranged from 1 to 10 years. Two had non-metastatic disease (gonadotroph PitNET and clinically functioning corticotroph PitNET), and two had metastatic disease (acidophil stem cell tumor and silent corticotroph tumor). Seven patients developed metastatic disease overall, including 3 that remained well-differentiated, 1 with features of acidophil stem cell tumor, 2 with sarcomatous transformation, and 1 with progressively increasing pleomorphism, mitotic count, and Ki-67 labeling index with each recurrence.

### Neuroimaging

**Fig. 1:**
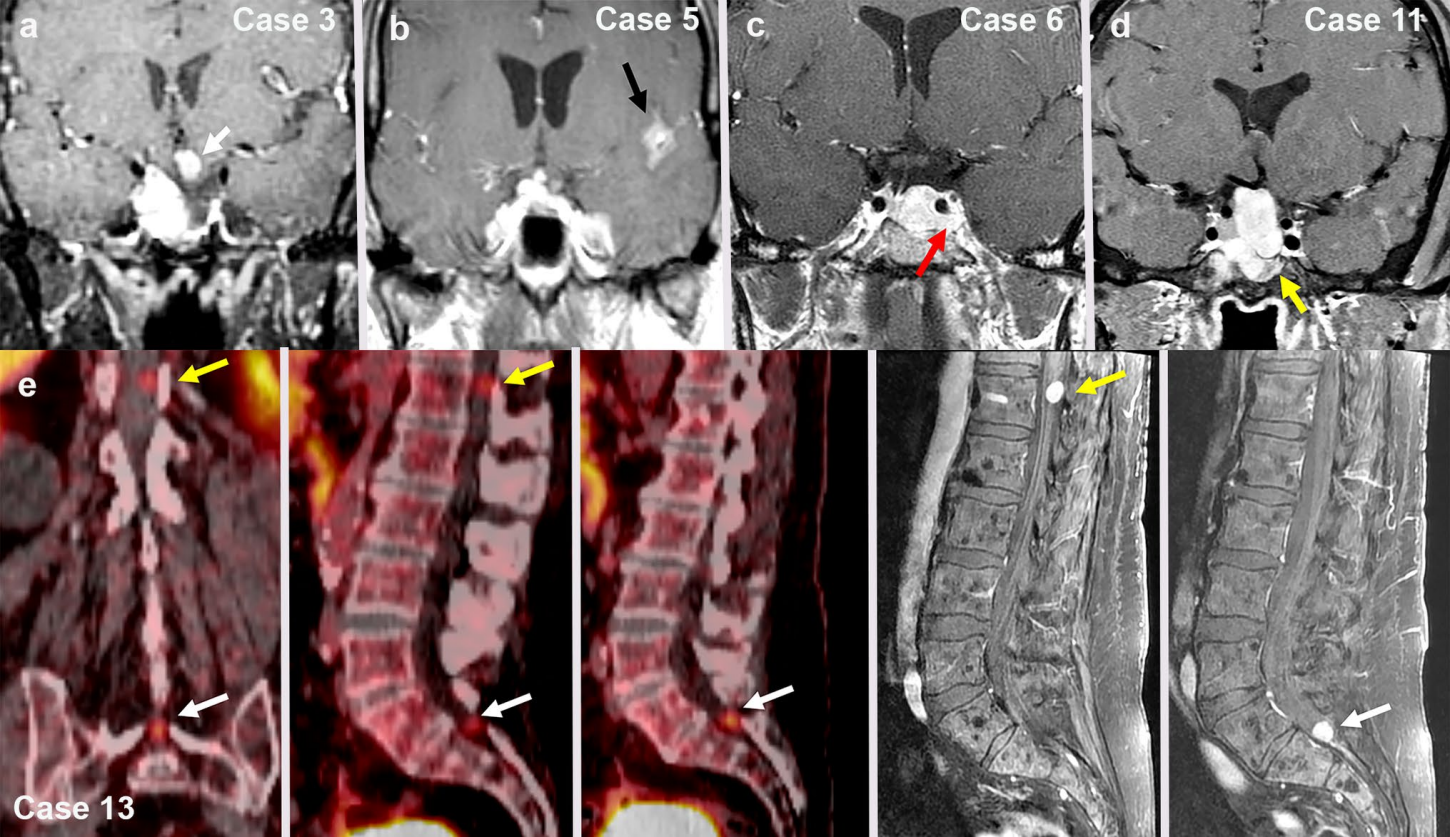
Examples of neuroimaging features on intracranial MRI (**a**, **d**) and spinal DOTATATE PET scan and MRI (**e**). **a** Separate infundibular enhancing nodule in patient 3 (white arrow). **b** Leptomeningeal enhancing nodule, representing CSF dissemination in patient 5 (black arrow). **c** Cavernous sinus invasion and expansion in patient 6 (red arrow). **d** Clival invasion in patient 11 (yellow arrow). **e** Two separate drop metastases representing CSF dissemination in case 13 (yellow and white arrows)

Magnetic resonance imaging (MRI) of the brain was available for 8 of 13 patients (Fig. 1). All tumors had a round or lobular contour, hypoenhancement compared to the native pituitary gland, and homogeneous T2 hypointense signal. All but one had cavernous sinus invasion (5 on the right, 1 bilateral, and 1 on the left). Seven patients had clival or central skull osseous invasion. Six had suprasellar nodules that were either contiguous or separate from the dominant sellar mass. None had infundibular stalk invasion, though the stalk was often deviated by the mass. PET imaging in case 2 revealed evidence of metastases involving the lung hilum, ovary, liver, and bone (not shown). DOTATATE PET scan in case 13 showed evidence of cervical spine bone metastasis and two drop metastases in the lumbosacral spinal cord (Fig. 1e).

### Pathology

The histopathologic, immunohistochemical, and molecular findings are summarized in Tables 2 and 3. Histologic subtypes of earliest reviewed PitNETs (Endocrine WHO 2022 guidelines) included acidophil stem cell tumor (*N* = 2), null cell tumor (*N* = 2), silent corticotroph tumor (*N* = 3), functioning corticotroph tumor (*N* = 1), gonadotroph tumor (*N* = 3), thyrotroph tumor (*N* = 1), and a lactotroph tumor in a male patient (*N* = 1).

**Table 2.**
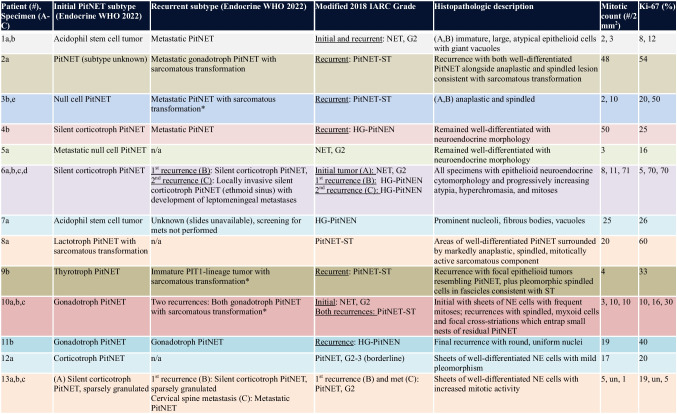
Tumor subtypes, grade, and histopathologic features

*History of radiation therapy. *HG-PitNEN* high-grade pituitary neuroendocrine neoplasm, *IARC* International Agency for Research on Cancer, *n/a* not applicable, *NET* neuroendocrine tumor, *PitNET* pituitary neuroendocrine tumor, *PitNET-ST* pituitary neuroendocrine tumor with sarcomatous transformation, *Un* unknown

**Table 3.**
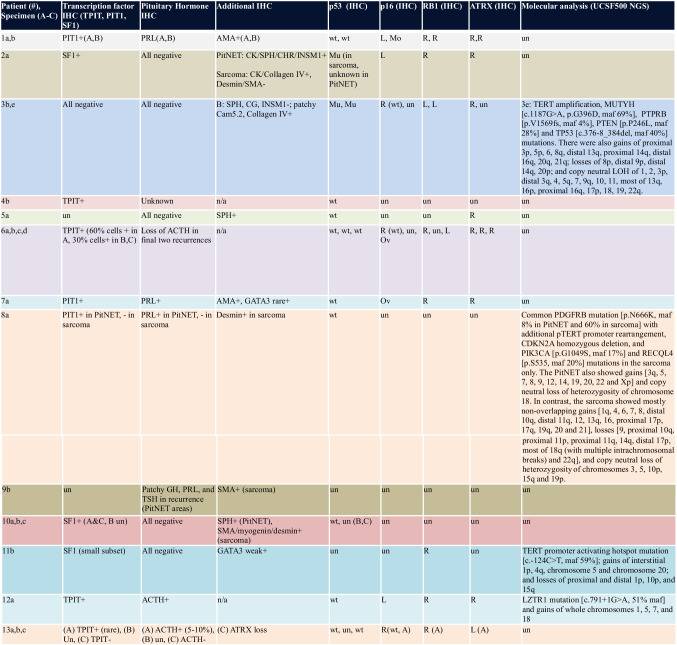
Immunohistochemical and molecular features

*AMA* anti-mitochondrial antigen, *IHC* immunohistochemistry, *L* loss of expression, *maf* mutant allele frequency, *maf* mutant allele frequency, *Mo* mosaic pattern of expression, *Mu* mutant, *NGS* next-generation sequencing, *Ov* overexpression, *R* retained expression, *Un* unknown, *wt* wildtype

**Fig. 2:**
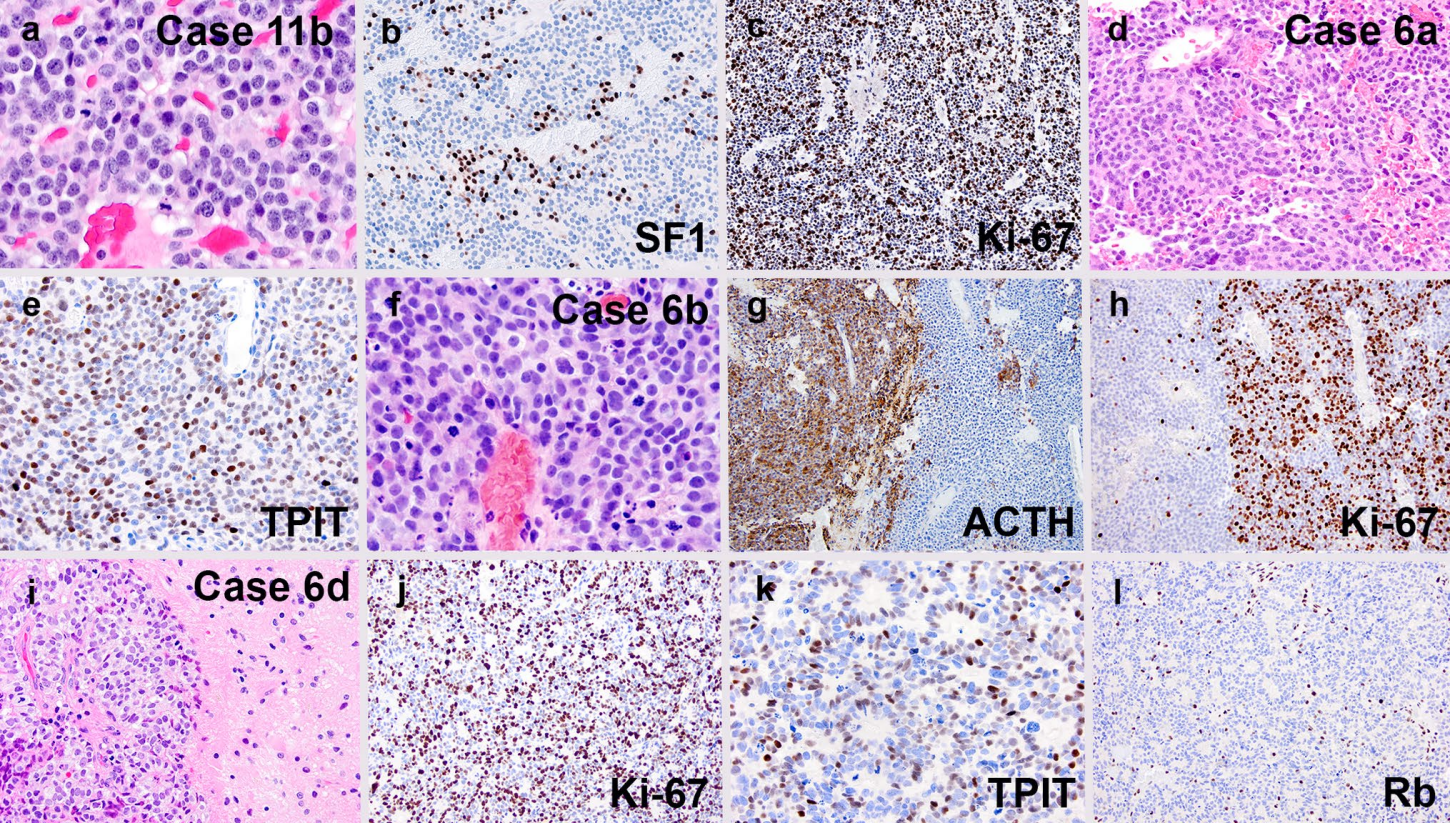
Examples of high-grade progression in cases 11 (**a**–**c**) and 6 (**d**–**l**). Case 11b showed similar features to a conventional gonadotroph tumor, but with up to 19 mitoses per 2 mm^2^ (**a**), less SF1 positivity than most (**b**), and a Ki-67 labeling index of roughly 40% (**c**). Case 6a initially presented as a silent corticotroph tumor with up to 8 mitoses per 2 mm.^2^ (**d**), diffuse ACTH expression (not shown), a Ki-67 labeling index of ~ 5% (not shown), and extensive TPIT positivity (**e**). At recurrence (Case 6b), there were similar areas to the original tumor, but focal areas showed a marked increase in mitotic count (**f**), loss of ACTH positivity (**g**, right half), and markedly increased Ki-67 labeling (**h**, right half). A subsequent resection (Case 6d) from a site of intracranial CSF dissemination showed brain invasion (**i**), markedly elevated Ki-67 labeling (**j**), lack of ACTH expression (not shown), partially retained TPIT positivity (**k**), and loss of Rb expression (**l**, note positivity in endothelial cells)

**Fig. 3:**
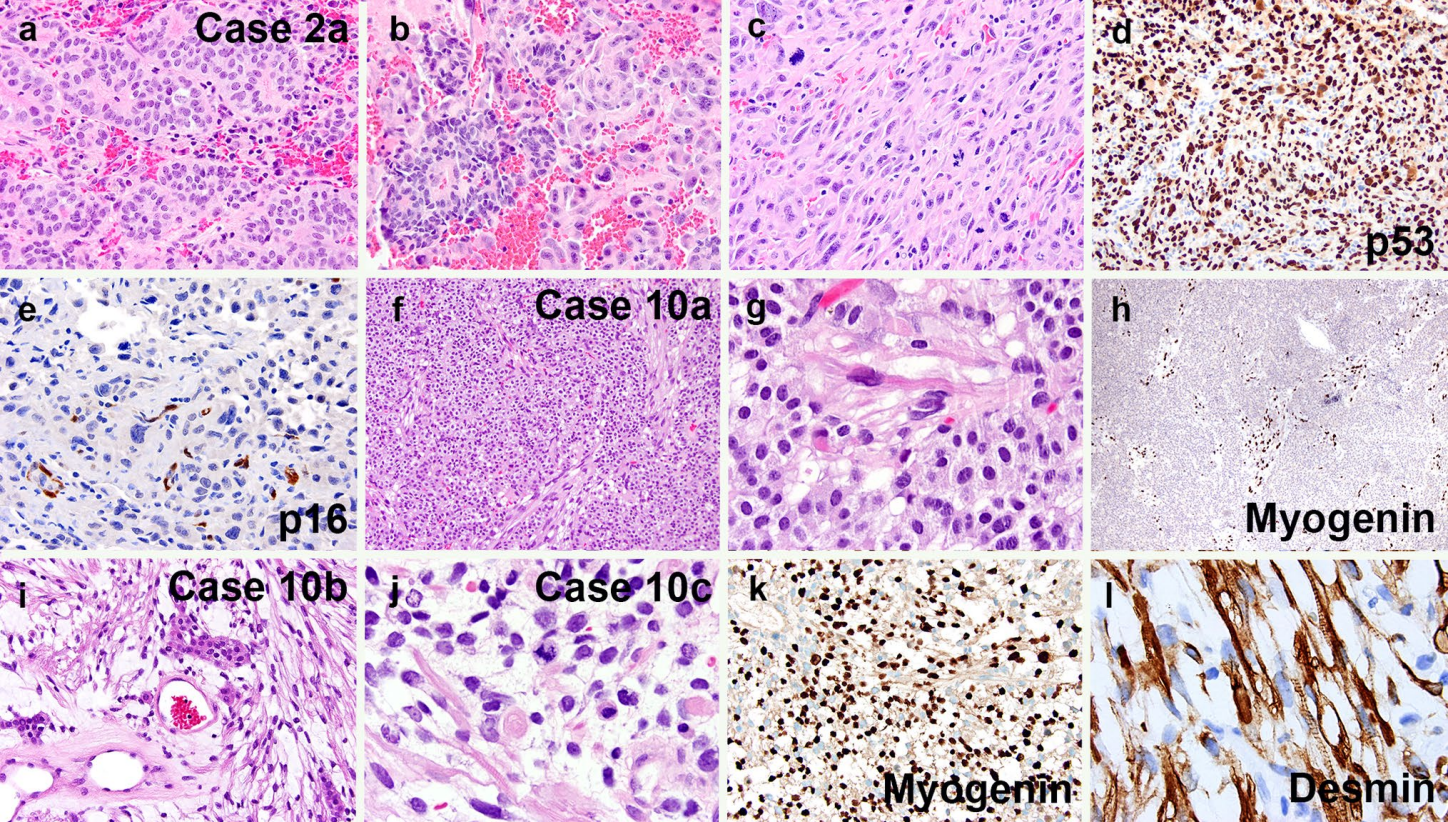
Examples of sarcomatous transformation in cases 2 and 10. Case 2a included both areas of conventional gonadotroph PitNET (**a**, **b** left half), and sarcomatous transformation (**b** right half, **c**). The latter showed p53 overexpression (**d**) and lack of p16 expression in tumor cells (**e**, note staining of endothelial cells). Case 10a started off as a gonadotroph tumor with scattered spindle-shaped rhabdomyoblastic cells (**f**, **g**), the latter of which were myogenin-positive (**h**). In subsequent recurrences, the rhabdomyosarcomatous component became increasingly predominant (**i**, **j**), as evidenced by both myogenin (**k**) and desmin (**l**) immunostains

**Fig. 4:**
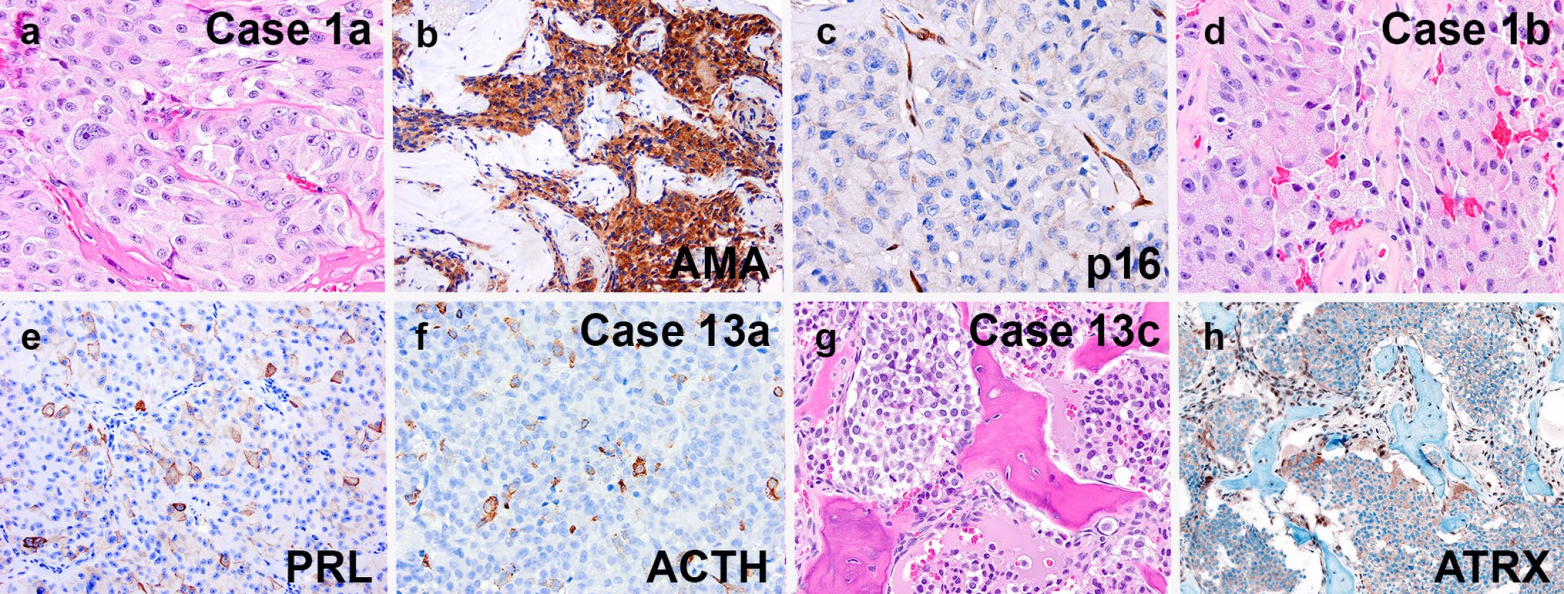
Examples of metastatic PitNET from cases 1 and 13. Case 1a was an acidophil stem cell tumor with marked cytologic atypia (**a**), patchy prolactin staining (not shown), and extensive positivity on the antimitochondrial antigen (AMA) stain (**b**). The p16 stain showed that the primary sellar tumor was negative (**c**, note internal positive control in blood vessels). A subsequent cervical lymph node resection showed similar histology (**d**) and patchy prolactin positivity (**e**) similar to the primary tumor. Case 13a was a silent corticotroph tumor with 5 mitoses per 2 mm.^2^ (not shown), a Ki-67 labeling index of 19% (not shown), and scattered immunoreactivity for ACTH (f) and TPIT (not shown). A subsequent bone metastasis specimen from the cervical vertebral body showed classic cytologic features of PitNET (g), but lack of any ACTH or TPIT expression (not shown). An immunostain for ATRX showed loss of expression in tumor cells (h, note staining of endothelial cells)

Using the modified IARC criteria, the primary tumors from three patients were IARC PitNET grade 2. All eventually metastasized. One of the grade 2 PitNETs remained grade 2 in both the initial specimen and the recurrence. However, 4 patients underwent progression to HG-PitNEN (Fig. 2). One patient with 17 mitoses per 2 mm^2^ and Ki-67 labeling index of 20% fell just shy of grade 3 PitNET and therefore was classified as borderline between IARC grades 2 and 3. Sarcomatous transformation was encountered in 5 cases, characterized by the presence of spindled cells with cytologic anaplasia, frequent mitoses, intercellular reticulin/collagen IV deposition, loss of pituitary transcription factors, and/or loss of pituitary hormones (Fig. 3). Of these, 3 tumors had been previously irradiated and 2 were de novo. CSF dissemination and/or hematogenous metastases were documented in 7 patients, including 5 with histologic confirmation (Fig. 4).

Two silent corticotroph PitNETs lost their ACTH expression in tumor recurrences or metastatic deposits, one also losing its TPIT immunoreactivity. The median Ki-67 labeling index for all cases was 25% (range 5–70%). Absence of tumoral p16 staining was seen in 3 cases (either loss of expression or lack of expression despite intact *CDKN2A* alleles), and p16 overexpression was seen in 2 cases. Strong diffuse p53 immunopositivity was present in 3 specimens from 2 patients. ATRX expression was lost in both the primary and metastatic tumors from one silent corticotroph PitNET (patient 13).

### Genetics

Next-generation sequencing analysis was performed in 4 tumors from 4 patients (Table 3). Three patients had *TERT* alterations (one *TERT* gene amplification, one *TERT* promoter rearrangement, and one *TERT* promoter activating hotspot mutation). In a patient with a null cell PitNET who went on to develop metastatic PitNET-ST (Specimen 3B), next-generation sequencing revealed a *TERT* amplification and *PTEN* and *TP53* mutations. In a patient with lactotroph PitNET who went on to develop a locally aggressive PitNET with sarcomatous transformation (Specimen 8A), sequencing of both the well-differentiated PitNET component and the sarcomatous components was performed. A *PDGFRB* mutation was common to both components. The sarcomatous component additionally harbored *TERT* promoter rearrangement, *CDKN2A* homozygous deletion, *PIK3CA* mutation, and aneuploidy. A patient with multiple and rapidly recurring gonadotroph PitNET (Specimen 11A) contained a *TERT* promoter mutation and many chromosomal copy number variations. Lastly, an *LZTR1* splice site mutation (c.791 + 1G > A), as well as gain of chromosomes 5 and 7, was seen in a Cushing syndrome-associated corticotroph tumor with borderline grade 2–3 features (Specimen 12A).

## Discussion

As evident from our data and those of prior investigators, PitNETs need not be metastatic to be highly clinically aggressive. In addition to the most common scenario of a locally invasive, but histologically well-differentiated and minimally proliferative PitNET, we identified rare cases with features of high-grade malignancy prior to any known metastasis. In our experience, local anaplastic transformation of PitNET occurs in one of two ways: sarcomatous transformation in a previously well-differentiated PitNET (with or without radiation therapy) or high-grade progression with retained epithelial cytology (increasing mitoses, Ki-67, and cytologic anaplasia, corresponding to IARC grade 3 PitNET or rarely, PitNEC).

While a formal grading system for PitNETs does not currently exist, the IARC system for grading neuroendocrine neoplasms elsewhere in the body may be of use when applied to pituitary neoplasms showing higher grade features. Namely, both grade 3 PitNET and PitNEC exist in rare cases. However, the IARC system also has some limitations. First, the distinction between these two high-grade tumor types was found to be subjective and challenging, leading us to lump these tumors into a bucket designation of “HG-PitNEN.” Second, we never encountered an example of high-grade pituitary neoplasm that would otherwise correspond to small cell neuroendocrine carcinoma, as defined elsewhere in the body. Third, as is already known, there are some PitNET subtypes that show considerably aggressive biology despite otherwise qualifying as a routine grade 1 tumor based on IARC criteria. Lastly, the IARC system does not account for PitNETs with sarcomatous transformation (hence our use of the term PitNET-ST). These tumors also show high-grade features, aggressive clinical features, and are distinct from conventional NEC. While some of the previously reported cases in the literature have been associated with prior radiation therapy and, as such, may represent radiation-induced sarcomas, the phenomenon of sarcomatous transformation is now also recognized in the absence of prior irradiation [19], as was also true of 2 of our current cases.

Examples of high-grade PitNEN have been rarely reported previously. Saeger et al. [11] reported two tumors with markedly elevated Ki-67 labeling indices, frequent mitoses, and *TP53* mutations (defined as nearly all tumor nuclei positive for p53). They similarly discussed how best to classify these tumors, including the incorporation of the IARC criteria. One case remained well-differentiated, despite high Ki-67 and mitoses, and could therefore qualify as PitNET G3. The other appeared poorly differentiated in addition to having high proliferative index and strong p53 staining in over half of tumor cells, prompting the authors to qualify it as a PitNEC. Pasquel et al. similarly reported a case of non-functioning PitNET with a Ki-67 labeling index of 30–80% [22] but no metastases, prompting them to propose a new term “carcinoma in situ.” Guo et al. reported a poorly differentiated, metastatic, corticotroph PitNET with a Ki-67 labeling index of 80%, as well as *TP53*, *ATRX*, and *PTEN* mutations [23].

The best nosology for aggressive PitNETs remains a matter of debate. In addition to the existing term of metastatic PitNET (previously pituitary carcinoma), we propose the use of HG-PitNEN (grade 3 PitNET and PitNEC) and PitNET-ST to account for current gaps or shortcomings in the nomenclature. Saeger and colleagues proposed to delineate all differentiated pituitary tumors with Ki-67 ≥ 50% and *TP53* mutation as PitNET G3, and all undifferentiated/poorly differentiated tumors as PitNEC, as well as abandoning the requirement for metastasis before considering a tumor as being overtly malignant [10].

All of our primary tumors had at least one worrisome feature within the sellar primary. These included histologic subtypes (by lineage and cytology) known to be aggressive, high mitotic count, and/or high Ki-67. Nevertheless, it is recognized that our series remains relatively small. As such, it is hard to make overarching generalizations regarding this issue, particularly when there are other metastatic and/or recurrent cases reported in the literature that did not otherwise show any clearly aggressive histologic features.

Relatively little is known about the genetic underpinnings of high-grade progression in PitNETs. Although epigenetic changes appear to be more common in PitNETs than classic genetic alterations, DNA methylation profiling is not a currently reliable and reproducible technique for distinguishing aggressive tumors from indolent counterparts [24]. Most PitNETs are without known mutations, with some exceptions: *GNAS* (somatotroph tumors), *USP8* (corticotroph tumors), and *ATRX* (corticotroph tumors). In our study, *TERT* alterations were present in 3 of 4 specimens tested. *TERT* promoter methylation has been previously reported in PitNET, but larger series are still needed to better determine the role of this marker [25]. Co-alteration of *TP53* and *RB1* are nearly universal in small cell lung cancer and very common in small cell NEC of other sites; this was similarly seen in the high-grade/sarcomatous areas from our cases 3 and 6. Large cell NEC generally has more heterogenous genetics with a lower fraction of cases demonstrating co-alteration of *TP53* and *RB1* compared with small cell NEC; this genetic heterogeneity likely reflects the morphologic variability of this group as well, with a fraction of cases likely representing G3 NET.

While no unifying histopathological or molecular feature has been identified to unequivocally distinguish biologically indolent PitNETs from aggressive counterparts, we identified a number of worrisome features that may prove useful in the early identification of aggressive tumors. These included aggressive subtype based on the currently standard lineage-based approach, histologic anaplasia (i.e., HG-PitNEN or sarcomatous histology), high mitotic count, high Ki-67 labeling index, loss of hormone and/or transcription factor expression, Rb loss, p16 absence or overexpression, *TP53* mutation, and presence of certain molecular alterations such as *TERT* alterations and homozygous *CDKN2A* inactivation. Larger studies are needed to further elucidate these individual variables with both univariate and multivariate analyses to better define their potential roles in future grading schemes.

## Data Availability

No datasets were generated or analysed during the current study.

## References

[1] Ezzat, S., Asa, S. L., Couldwell, W. T., Barr, C. E., Dodge, W. E., Vance, M. L., & McCutcheon, I. E. (2004). The prevalence of pituitary adenomas: a systematic review. *Cancer*, *101*(3), 613–619. https://doi-org.ucsf.idm.oclc.org/10.1002/cncr.20412 10.1002/cncr.20412 15274075

[2] Ostrom, Q. T., Gittleman, H., Truitt, G., Boscia, A., Kruchko, C., & Barnholtz-Sloan, J. S. (2018). CBTRUS Statistical Report: Primary Brain and Other Central Nervous System Tumors Diagnosed in the United States in 2011-2015. *Neuro-oncology, 20*(suppl_4), iv1–iv86. https://doi-org.ucsf.idm.oclc.org/10.1093/neuonc/noy131 10.1093/neuonc/noy131 PMC6129949 30445539

[3] Raverot, G., Burman, P., McCormack, A., Heaney, A., Petersenn, S., Popovic, V., Trouillas, J., Dekkers, O. M., & European Society of Endocrinology (2018). European Society of Endocrinology Clinical Practice Guidelines for the management of aggressive pituitary tumours and carcinomas. *European journal of endocrinology*, *178*(1), G1–G24. https://doi-org.ucsf.idm.oclc.org/10.1530/EJE-17-0796 10.1530/EJE-17-0796 29046323

[4] Trouillas, J., Jaffrain-Rea, M. L., Vasiljevic, A., Dekkers, O., Popovic, V., Wierinckx, A., McCormack, A., Petersenn, S., Burman, P., Raverot, G., & Villa, C. (2020). Are aggressive pituitary tumors and carcinomas two sides of the same coin? Pathologists reply to clinician's questions. *Reviews in endocrine & metabolic disorders*, *21*(2), 243–251. https://doi-org.ucsf.idm.oclc.org/10.1007/s11154-020-09562-9 10.1007/s11154-020-09562-9 32504268

[5] Kontogeorgos, G., Thodou, E., Osamura, R. Y., & Lloyd, R. V. (2022). High-risk pituitary adenomas and strategies for predicting response to treatment. *Hormones (Athens, Greece)*, *21*(1), 1–14. https://doi-org.ucsf.idm.oclc.org/10.1007/s42000-021-00333-y 10.1007/s42000-021-00333-y 35061210

[6] Trouillas, J., Roy, P., Sturm, N., Dantony, E., Cortet-Rudelli, C., Viennet, G., Bonneville, J. F., Assaker, R., Auger, C., Brue, T., Cornelius, A., Dufour, H., Jouanneau, E., François, P., Galland, F., Mougel, F., Chapuis, F., Villeneuve, L., Maurage, C. A., Figarella-Branger, D., … Tabarin, A. (2013). A new prognostic clinicopathological classification of pituitary adenomas: a multicentric case-control study of 410 patients with 8 years post-operative follow-up. *Acta neuropathologica*, *126*(1), 123–135. https://doi-org.ucsf.idm.oclc.org/10.1007/s00401-013-1084-y 10.1007/s00401-013-1084-y 23400299

[7] Trouillas, J., Burman, P., McCormack, A., Petersenn, S., Popovic, V., Dekkers, O., & Raverot, G. (2018). Aggressive pituitary tumours and carcinomas: two sides of the same coin?. *European journal of endocrinology*, *178*(6), C7–C9. https://doi-org.ucsf.idm.oclc.org/10.1530/EJE-18-0250 10.1530/EJE-18-0250 29588294

[8] Asa S, Perry A. Tumors of the Pituitary Gland. AFIP Atlas of Tumor and Non-tumor Pathology, 5th series Fascicle 1. Arlington, VA: ARP Press; 2020.

[9] Rindi, G., Klimstra, D. S., Abedi-Ardekani, B., Asa, S. L., Bosman, F. T., Brambilla, E., Busam, K. J., de Krijger, R. R., Dietel, M., El-Naggar, A. K., Fernandez-Cuesta, L., Klöppel, G., McCluggage, W. G., Moch, H., Ohgaki, H., Rakha, E. A., Reed, N. S., Rous, B. A., Sasano, H., Scarpa, A., … Cree, I. A. (2018). A common classification framework for neuroendocrine neoplasms: an International Agency for Research on Cancer (IARC) and World Health Organization (WHO) expert consensus proposal. *Modern pathology: an official journal of the United States and Canadian Academy of Pathology, Inc*, *31*(12), 1770–1786. https://doi-org.ucsf.idm.oclc.org/10.1038/s41379-018-0110-y 10.1038/s41379-018-0110-y PMC6265262 30140036

[10] Saeger, W., Mawrin, C., Meinhardt, M., Wefers, A. K., & Jacobsen, F. (2022). Two Pituitary Neuroendocrine Tumors (PitNETs) with Very High Proliferation and TP53 Mutation - High-Grade PitNET or PitNEC?. *Endocrine pathology*, *33*(2), 257–262. https://doi-org.ucsf.idm.oclc.org/10.1007/s12022-021-09693-y 10.1007/s12022-021-09693-y PMC9135791 34669159

[11] Mete, O., & Lopes, M. B. (2017). Overview of the 2017 WHO Classification of Pituitary Tumors. *Endocrine pathology*, *28*(3), 228–243. https://doi-org.ucsf.idm.oclc.org/10.1007/s12022-017-9498-z 10.1007/s12022-017-9498-z 28766057

[12] Knosp, E., Steiner, E., Kitz, K., & Matula, C. (1993). Pituitary adenomas with invasion of the cavernous sinus space: a magnetic resonance imaging classification compared with surgical findings. *Neurosurgery*, *33*(4), 610–618. https://doi-org.ucsf.idm.oclc.org/10.1227/00006123-199310000-00008 10.1227/00006123-199310000-00008 8232800

[13] Hardy J. Transsphenoidal surgery of hypersecreting pituitary tumours. In: Kohler PO, Ross GT, editors. Diagnosis and Treatment of Pituitary Tumours. Int. Congress Series No. 303. Amsterdam: Exerpta Medica; 1973. p. 179–98

[14] Raverot, G., Dantony, E., Beauvy, J., Vasiljevic, A., Mikolasek, S., Borson-Chazot, F., Jouanneau, E., Roy, P., & Trouillas, J. (2017). Risk of Recurrence in Pituitary Neuroendocrine Tumors: A Prospective Study Using a Five-Tiered Classification. *The Journal of clinical endocrinology and metabolism, 102*(9), 3368–3374.https://doi-org.ucsf.idm.oclc.org/10.1210/jc.2017-00773 10.1210/jc.2017-00773 28651368

[15] Lelotte, J., Mourin, A., Fomekong, E., Michotte, A., Raftopoulos, C., & Maiter, D. (2018). Both invasiveness and proliferation criteria predict recurrence of non-functioning pituitary macroadenomas after surgery: a retrospective analysis of a monocentric cohort of 120 patients. *European journal of endocrinology*, *178*(3), 237–246.https://doi-org.ucsf.idm.oclc.org/10.1530/EJE-17-0965 10.1530/EJE-17-0965 29259039

[16] Asioli, S., Righi, A., Iommi, M., Baldovini, C., Ambrosi, F., Guaraldi, F., Zoli, M., Mazzatenta, D., Faustini-Fustini, M., Rucci, P., Giannini, C., & Foschini, M. P. (2019). Validation of a clinicopathological score for the prediction of post-surgical evolution of pituitary adenoma: retrospective analysis on 566 patients from a tertiary care centre. *European journal of endocrinology*, *180*(2), 127–134. https://doi-org.ucsf.idm.oclc.org/10.1530/EJE-18-0749 10.1530/EJE-18-0749 30481158

[17] Terry, M., Reis, G., Horvai, A., Pekmezci, M., & Perry, A. (2023). Sarcomatous Transformation of a Medically Treated Lactotroph Pituitary Neuroendocrine Tumor?. *Endocrine pathology, 34*(1), 161–163.https://doi-org.ucsf.idm.oclc.org/10.1007/s12022-023-09757-1 10.1007/s12022-023-09757-1 PMC10011270 36826690

[18] Duncan, V. E., Nabors, L. B., Warren, P. P., Conry, R. M., Willey, C. D., Perry, A., Riley, K. O., & Hackney, J. R. (2016). Primary Sellar Rhabdomyosarcoma Arising in Association With a Pituitary Adenoma. *International journal of surgical pathology*, *24*(8), 753–756. https://doi-org.ucsf.idm.oclc.org/10.1177/1066896916658955 10.1177/1066896916658955 27422470

[19] Rindi, G., Mete, O., Uccella, S., Basturk, O., La Rosa, S., Brosens, L. A. A., Ezzat, S., de Herder, W. W., Klimstra, D. S., Papotti, M., & Asa, S. L. (2022). Overview of the 2022 WHO Classification of Neuroendocrine Neoplasms. *Endocrine pathology*, *33*(1), 115–154. https://doi-org.ucsf.idm.oclc.org/10.1007/s12022-022-09708-2 10.1007/s12022-022-09708-2 35294740

[20] Louis, D. N., Perry, A., Wesseling, P., Brat, D. J., Cree, I. A., Figarella-Branger, D., Hawkins, C., Ng, H. K., Pfister, S. M., Reifenberger, G., Soffietti, R., von Deimling, A., & Ellison, D. W. (2021). The 2021 WHO Classification of Tumors of the Central Nervous System: a summary. *Neuro-oncology*, *23*(8), 1231–1251. https://doi-org.ucsf.idm.oclc.org/10.1093/neuonc/noab106 10.1093/neuonc/noab106 PMC8328013 34185076

[21] Lucas, C. G., Villanueva-Meyer, J. E., Whipple, N., Oberheim Bush, N. A., Cooney, T., Chang, S., McDermott, M., Berger, M., Cham, E., Sun, P. P., Putnam, A., Zhou, H., Bollo, R., Cheshier, S., Poppe, M. M., Fung, K. M., Sung, S., Glenn, C., Fan, X., Bannykh, S., … Solomon, D. A. (2020). Myxoid glioneuronal tumor, PDGFRA p.K385-mutant: clinical, radiologic, and histopathologic features. *Brain pathology (Zurich, Switzerland)*, *30*(3), 479–494. https://doi-org.ucsf.idm.oclc.org/10.1111/bpa.12797 10.1111/bpa.12797 PMC7780370 31609499

[22] Pasquel, F. J., Vincentelli, C., Brat, D. J., Oyesiku, N. M., & Ioachimescu, A. G. (2013). Pituitary carcinoma in situ. *Endocrine practice: official journal of the American College of Endocrinology and the American Association of Clinical Endocrinologists*, *19*(3), e69–e73. https://doi-org.ucsf.idm.oclc.org/10.4158/EP12351.CR 10.4158/EP12351.CR 23425649

[23] Guo, F., Wang, G., Wang, F., Xu, D., & Liu, X. (2018). Identification of Novel Genes Involved in the Pathogenesis of an ACTH-Secreting Pituitary Carcinoma: A Case Report and Literature Review. *Frontiers in oncology*, *8*, 510. https://doi-org.ucsf.idm.oclc.org/10.3389/fonc.2018.00510 10.3389/fonc.2018.00510 PMC6232249 30460199

[24] Guaraldi, F., Morandi, L., Zoli, M., Mazzatenta, D., Righi, A., Evangelisti, S., Ambrosi, F., Tonon, C., Giannini, C., Lloyd, R. V., & Asioli, S. (2022). Epigenomic and somatic mutations of pituitary tumors with clinical and pathological correlations in 111 patients. *Clinical endocrinology*, *97*(6), 763–772. https://doi-org.ucsf.idm.oclc.org/10.1111/cen.14827 10.1111/cen.14827 PMC9828656 36161330

[25] Miyake, Y., Adachi, J. I., Suzuki, T., Mishima, K., Araki, R., Mizuno, R., & Nishikawa, R. (2019). TERT promoter methylation is significantly associated with TERT upregulation and disease progression in pituitary adenomas. *Journal of neuro-oncology*, *141*(1), 131–138. https://doi-org.ucsf.idm.oclc.org/10.1007/s11060-018-03016-8 10.1007/s11060-018-03016-8 30392088

